# Leprosy in children and adolescents under 15 years old in an urban centre in Brazil

**DOI:** 10.1590/0074-02760160002

**Published:** 2016-06

**Authors:** Selton Diniz Santos, Gerson Oliveira Penna, Maria da Conceição Nascimento Costa, Marcio Santos Natividade, Maria Glória Teixeira

**Affiliations:** 1 Universidade Federal da Bahia, Instituto de Saúde Coletiva, Salvador, BA, Brasil; 2 Fundação Oswaldo Cruz, Escola Fiocruz de Governo, Brasília, DF, Brasil

**Keywords:** leprosy, children, epidemiology, urban transmission, spatial distribution

## Abstract

This original study describes the intra-urban distribution of cases of leprosy in residents under 15 years old in Salvador, Bahia, Brazil; the study also identifies the environment in which *Mycobacterium leprae* is being transmitted. The cases were distributed by operational classification, clinical forms, type of contact and the addresses were geo-referenced by neighborhood. Between 2007 and 2011, were reported 145 cases of leprosy in target population living in Salvador, corresponding to detection rates of 6.21, 6.14, 5.58, 5.41 and 6.88/100,000 inhabitants, respectively. The spatial distribution of the disease was focal. Of the 157 neighborhoods of Salvador, 44 (28.6%) notified cases of leprosy and in 22 (50%) of these were detected more than 10 cases per 100,000 inhabitants. The infectious forms were found in 40% of cases. Over 90% of cases had been living in Salvador for more than five years. Overall, 52.6% reported having had contact with another infected individual inside the household and 25% in their social circle. In Salvador, *M. leprae* transmission is established. The situation is a major concern, since transmission is intense at an early age, indicating that this endemic disease is expanding and contacts extend beyond individual households.

Lessons learned through efforts to eliminate leprosy show that the reduction in treatment time made possible by multidrug therapy has been insufficient to achieve the goal proposed by the World Health Organization in 1991 (Lockwood et al. 2014). Therefore, in countries in which leprosy is endemic, the condition remains a significant health issue in terms of magnitude, transcendence and vulnerability. Lack of any specific and effective vaccine against this disease has hampered control actions that continue to focus exclusively on reducing the sources of infection by treating patients (WHO 2016).

Since the end of the 20th century, leprosy has continued to expand in Brazil, with the country now in second place behind only India in the number of cases detected. Brazil accounts for over 91% of leprosy notifications in Latin America (Penna et al. 2009, WHO 2016). One of the determining factors in this expansion was the intense migration flow of rural populations to urban areas in search of better life conditions in the most economically important cities of the country. These migratory movements favored the dissemination of *Mycobacterium leprae* to cities where the disease had previously been absent or where the number of cases had been minimal. Indeed, the rapid urbanisation of Brazil changed the occurrence and distribution pattern of several endemic diseases, including leprosy, the agent of which had been restricted to rural regions until the first half of the 20th century (Kerr et al. 2004, Aagaard-Hansen et al. 2010,Murto et al. 2012).

Due to the long incubation period of this disease it is more common in adults. Nevertheless, in endemic locations, children and adolescents, theoretically considered the group most resistant to infection, become vulnerable as a result of being continuously exposed to foci of active transmission from a very young age (Fine 1982, 1992). Therefore, evaluating the occurrence of leprosy in children under 15 years old is crucial, since this reflects the intensity of propagation of the infectious agent, as well as being important for monitoring the epidemiological impact of the control actions implemented.

Maintenance of the epidemiological cycle of leprosy is attributed to close, prolonged contact between susceptible healthy individuals and untreated multibacillary cases (Mira et al. 2003, 2004), with household transmission being the most significant in terms of public health (WHO 2010). Nevertheless, unlike rural areas, living conditions and social spaces (work, school and the neighborhood) in modern urban centres encourage intense, continuous contact between human beings. It is therefore reasonable to speculate that the current increase in the number of detected cases of leprosy in cities may also be a result of the exposure of susceptible individuals to untreated infected patients even outside the household (Van Beers et al. 1999). In Brazil, the rate detection of leprosy in children and adolescents is high, principally in urban agglomerations with poor living conditions, where the risk of the disease is greater (Ferreira et al. 2007, Penna et al. 2009, WHO 2015). Therefore, it is relevant to evaluate the extent to which leprosy is being transmitted in environments other than households in order to gain understanding on the factors involved in this process.

Bahia (BA) is one of the Brazilian states in which leprosy is most endemic, with a general detection rate of 20.5/100,000 inhabitants and of 6.4/100,000 under 15 years of age, in 2015 (SESAB 2016). Bahia’s capital city, Salvador, is the most densely populated municipality in the state and the overall detection rate of leprosy in the city was 15.6 cases and 6.0 per 100,000 inhabitants, respectively, to the total population and under 15 years of age, in that same year (SMS 2016).

The objective of this original study was to describe the intra-urban distribution of cases of leprosy in under 15 years of age residents in Salvador, BA, Brazil and to identify the environment in which *M. leprae* is being transmitted in this age group.

## MATERIALS AND METHODS

It was a confirmed cases study of leprosy in children under 15 years of age residents in Salvador, diagnosed between 2007 and 2011. The population of this city was 2,710,968 inhabitants in 2011 (MS/Datasus 2011). An ecological study also was conducted with these leprosy cases, considering the neighborhood as the unit of analysis.

The data on registered cases of this disease were obtained from the Information System on Notifiable Diseases (SINAN) of the Health Department of the state. After excluding cases lacking identification or address and those referring to individuals who did not live in Salvador, the following data were obtained: name, sex, age and address, date of diagnosis, operational classification and clinical form of the disease. These classifications are done by the physicians in the Care Services according the criteria of Ministry of Health (MS/SVS 2014). During October and November 2012, interviews were held at the homes of cases, with their parents or guardians or with the individual if he/she was > 18 years at the time of the interview. Four university-level healthcare professionals with experience in the field of public health conducted the interviews. The interviewers had been previously trained on aspects related to the disease, the objectives and methodology of the study, and the care to be taken with regard to confidentiality and respect, considering that these were children and adolescents and that leprosy is a stigmatising disease.

After the individual had read and signed the informed consent form, an interview was conducted and recorded. The interview followed a script, with questions on the individual’s migratory history and any contact between the case and another person with the disease. The data obtained were recorded on a structured questionnaire. The cases’ addresses were geo-referenced using the Global Position System. The addresses were initially geo-referenced according to census tracts and then aggregated by neighborhood. Patterns of spatial distribution were identified by visually inspecting thematic maps constructed using the ArcView software program, version 3.1.

Individuals with leprosy living in Salvador for at least five years prior to diagnosis of the disease were considered to have acquired the infection in this city. If the individual had migrated to Salvador less than five years prior to diagnosis, the place of transmission was defined as having been outside this municipality. If there was information on the existence of another case of leprosy in the household, contact was considered as having occurred within the household. Contact was considered social when the participant reported no other case of leprosy in the household but referred to another case of the disease in the neighborhood or among close friends with known contact between that individual and the case under investigation. The type of contact was considered both household and social when both forms of contact were reported. Finally, contact was defined as unknown when no information was available on any contact between the case and any other leprosy patient.

The variables analysed were sex, age group (0-4, 5-9 and 10-14 years); operational classification [multibacillary (MB) or paucibacillary (PB)]; clinical form [indeterminate, tuberculoid, boderline or lepromatous (Virchowian)]; type of contact (household, social, both household and social, or unknown), neighborhood of residence in Salvador; and having lived in another city up to five years before living in Salvador.

The spatial distribution included the 157 neighborhoods. Following, the neighborhoods also were grouped by the 12 sanitary districts (SD) of Salvador. Sanitary district is the smaller population or geographical area used in the planning and management process of the Health System (SMS 2009). The detection rate in under 15 was calculated by neighborhood of residence and then classified in accordance with the parameters adopted by Ministry of Health (MS 2009) as low (< 0.50/100,000 inhabitants); intermediate (0.5-2.49/100,000 inhabitants); high (2.5-4.99/100,000 inhabitants); very high (5.0-9.99/100,000 inhabitants) and hyperendemic (> 10.0/100,000 inhabitants).

The other variables were described using proportions. Fisher’s exact test was used to verify the existence of differences in the distribution of age, sex and the type of contact. Significance was established at p < 0.05. Data processing and analysis were conducted using the Stata statistical software program, version 12.1 (Copyright 1985-2011 StataCorp LP, College Station, TX).

The Human Research Ethics Committee of the Collective Health Institute, Federal University of Bahia approved this study, Protocol number 119.265/2012. The parents of the children provided Informed Consent written, according the Resolution 466/2012 of the Conselho Nacional de Ética em Pesquisa em Seres Humanos (CONEP/Brazil).

## RESULTS

Between 2007 and 2011, 145 cases of leprosy were reported in SINAN in children and adolescents under 15 years of age living in Salvador, corresponding to detection rates of 6.21, 6.14, 5.58, 5.41 and 6.88/100,000 inhabitants, respectively, for each year of the series evaluated in this study. Table I shows the characteristics of the study population according to the operational classification of the disease. The incidence was found to be higher in girls (51.7%) and in the 10-14 years age group (59.3%). In addition, the most common clinical form of the disease was the tuberculoid form (44.1%). Clinical classification was unavailable in only 5.5% of cases. Children under five years of age constituted 10.3% of cases. Among the PB cases (60.7%), there was also a greater proportion of girls (56.8%), adolescents in the 10-14 years age group (48.9%) and the tuberculoid clinical form (69.3%). In the group of MB cases, which represented 39.3% of all the cases analysed, there was a predominance of adolescents in the 10-14 years age group (75.4%), boys (56.3%) and the borderline clinical form of the disease (59.7%). The lepromatous clinical form of the disease was found in cases aged between eight-14 years, with the highest frequency in those of 11 years of age (30%). The differences found between the PB and MB forms were statistically significant to age group and clinical form (p < 0.05).

**TABLE I:** Numbers and percentage of leprosy cases in children < 15 years old by operational classification of the disease according to sex, age group and clinical form (n = 145). Salvador, Bahia, Brazil, 2007-2011

Variables	Classification				Total	

	Paucibacillary		Multibacillary			
		
	n = 88	(%)	n = 57	(%)	n (%)	
Sex						
Female	50	56.8	25	43.7	75	51.7
Male	38	43.2	32	56.3	70	48.3
Age group (year)						
0-4	12	13.6	3	5.3	15	10.3
5-9	33	37.5	11	19.3	44	30.3
10-14	43	48.9	43	75.4	86	59.3
Clinical form						
Indeterminate	15	17.0	1	1.7^1^	16	11.0
Tuberculoid	61	69.3	3	5.3	6	44.1
Borderline	7	8.0	34	59.7	41	28.3
Lepromatous	-	-	16	28.0	16	11.0
Not classified	5	5.7	3	5.3	8	5.6

n = number of individuals; ^1^p < 0,05.

Of the 145 reported leprosy cases registered in the SINAN, 76 (52.4%) were successfully located and interviewed. In the other cases, either they were unable to be found or their parents did not consent to the interview. Of the 76 cases interviewed, only eight (10.5%) had a history of migration. Of these, four cases had lived outside Salvador for a period ≤ 5 years immediately preceding diagnosis. Three of these individuals had come from urban areas of other states.

Of the 157 neighborhoods of Salvador (Figure), 113 (69.4%) did not report cases of leprosy during the study period, and were classified as of low endemicity. The 44 (28.6%) neighborhoods with cases reported were classified as flollows: one (0.63%) average endemicity, nine (5.73%) high endemicity, 12 (7.64%) very high, and 26 (56%) hyperendemic. The majority of these hyperendemic neighborhoods were located in the SD of São Caetano/Valéria, Cajazeiras, Subúrbio Ferroviário and Itapuã.

**Figure:**
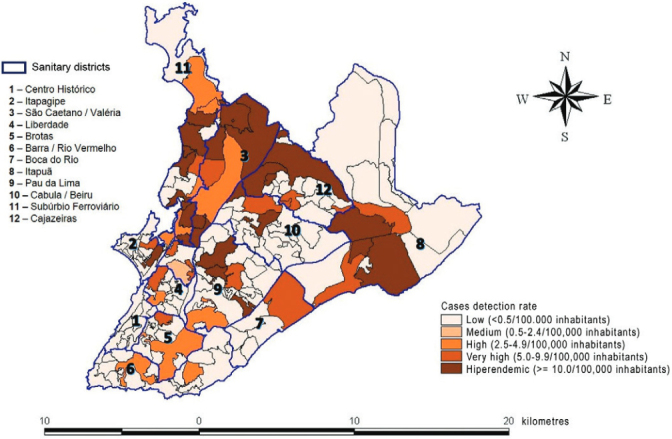
Distribution of the detection rate of leprosy cases in children < 15 years old by neighborhoods according to the parameters of risk level. Salvador, Bahia, Brazil, 2007-2011.

Table II shows the sociodemographic characteristics of the cases of leprosy in children under 15 years of age according to the type of contact the patient had with this disease. According to the results of this study, 52.6% of the participants reported having had contact with another case of leprosy in their home (household contact). On the other hand, 25% reported that contact with the disease occurred socially (in the neighborhood, at school or through a close friend), 5.2% mentioned having had both types of contact (household and social) and 17.1% reported not knowing or not having had any contact with anyone with the disease. There is statistically significant difference between the two types of contact according to sex, with household contact being more common among girls (75%) and social contact more common among boys (52.6%) (p < 0.005).

**TABLE II:** Numbers and percentage of leprosy cases in children < 15 years old by the type of contact according to sex and age group (n = 76). Salvador, Bahia, Brazil, 2007-2011

Variables	Contact type									Total		

	Household			Social		Household and social		Unknown				
				
	n = 40		(%)	n = 19	(%)	n = 4	(%)	n = 13	(%)	n = 76	(%)	
Sex												
Female	21	52.5		9	47.4	3	75.0	9	69.2	42	55.3	
Male	19	47.5		10	52.6	1	25.0	4	30.8	34	44.7	
Age group (year)												
0-4	9	23.1		1	5.3	-	-	1	7.7	15	10.3	
5-9	12	30.8		6	31.6	2	50	3	23.1	44	30.3	
10-14	18	46.1		12	63.1	2	50	9	69.2	86	59.3	

## DISCUSSION

The present study shows that in Salvador, the third largest urban center in Brazil, transmission of *M. leprae* is intense. This fact is revealed not only by the high detection rates of cases of leprosy in children and adolescents under 15 years of age, but principally because almost all the participants in this study were already living in this city when they acquired the infection. Furthermore, over 10% of cases were diagnosed in the first four years of life. The situation of hyperendemicity in this city is evident, since the detection rate of this disease in the general population has never fallen below 10.6/100,000 inhabitants since 2001.

In 2010, 6.9/100,000 inhabitants under 15 years old were affected, that is almost three times the maximum limit considered by the World Health Organization as high endemicity. This highlights the severity of the epidemiological situation of this disease in Salvador.

The fact that children under 15 years old are being infected by this disease at an early age makes this scenario even more of a concern, since it indicates that a significant proportion of cases are not being detected or treated opportunely to sterilise the sources of the infection and prevent or reduce transmission of the agent. As leprosy in Salvador has occurred in aggregates in a restricted number of neighborhoods, implementation of early detection and the timely treatment of cases would be expected since this scenario should facilitate intervention by healthcare services. In fact, services could focus on specific activities within circumscribed territory while intensifying surveillance and control actions. Nevertheless, the scenario seen here suggests that the action of leprosy control program is not sufficient to reduce the sources of infection and, consequently, to protect the population, principally the children. There is gap in the knowledge in the leprosy transmission process and it is important to evidence that the actions of prevention of leprosy still are limited to promote the elimination of this disease. So, nowadays the prevention continue centred only in individual treatment, the poliquimioterapy, that did not produced the effectiveness awaited (Lockwood et al. 2014).

These results strongly suggest that the transmission of leprosy in Salvador is not restricted to the household. Indeed, in over 40% of cases either the contact was social or the environment in which the individual had been infected was unknown, giving strength to the hypothesis that the role of extra-household contacts in the transmission of this disease within cities is important, as it has also been demonstrated by other authors (Moet et al. 2006, Hoeven 2008, Moura et al. 2013 **)** . The rise in the incidence with the increasing age of the child is the result of the greater probability of infection as the time of exposure to the sources of infection increases, as has already been reported in the literature (Selvasekar et al. 1999, Ferreira et al. 2007). Nevertheless, the detection of cases in children under four years of age cannot be considered trivial, since it reached 10.3%, representing yet more evidence of the active and autochthonous transmission of *M. leprae* in this city. The occurrence of leprosy in such a high proportion as that found in this study shows that in certain geographical spaces of Salvador, very young children are intensely exposed to the agent.

In general, PB is the more common type in under-15s and characterises the initial and transitory stages of the disease (Lana et al. 2007, Oliveira 2008, Santos et al. 2008), although an inversion may occur in this pattern in hyperendemic areas (Ferreira et al. 2007). In Salvador, the predominant form was truly PB (60.7%); however, around 40% of the cases were of the MB type, which is responsible for maintaining the epidemiological cycle of the agent.

The high frequency of the tuberculoid clinical form of the disease (44.1%), which, in general, is expressed in individuals with greater cell immunity against *M. leprae*, has been considered an indication that the endemic disease is in expansion (Lombardi et al. 1990). In this type of situation, it is necessary to intensify the search for and examination of contacts (Oliveira 2008) to identify possible sources of the infection with a view to implementing treatment at an early stage and, consequently, reducing further occurrences.

The risk of acquiring the disease is greater among household contacts in relation to other types of contact (George et al. 1990,Fine et al. 1997, Vijayakumaran et al. 1998, Moet et al. 2006). Additionally, study with Vietnamese and Brazilian families showed an association of genetic factors that rendered some individuals more susceptible to clinically expressing *M. leprae* infections (Mira et al. 2004). Nevertheless, based on the well-established fact that the disease has a long incubation period and a broad spectrum of clinical types, being a household contact of a leprosy case is a very important factor in acquiring the infection and, consequently, the disease. Anyway, since the majority of known sources of infection are in the household, this characteristic of the epidemiological cycle reduces the range involved when actively searching for cases. Nonetheless, it should be considered that 25% of the cases reported social contact (in the same neighborhood or during other opportunities of social, work or school-related contact). The greatest hurdles to developing control actions refer to the individuals who are unable to provide any information on their source of contact. In these situations, the source of infection may indeed be unknown or the information may not be readily available because of the social stigma associated with the disease, which often prevents patients from revealing the existence of the disease in the family.

Focal and heterogeneous distribution, which is characteristic of leprosy (Magalhães & Rojas 2005, Penna et al. 2009), was also present in Salvador, where aggregates were found to be concentrated in certain neighborhoods. Leprosy has historically been associated with poverty (Kerr et al. 2004, Magalhães & Rojas 2005) and in these neighborhoods the socioeconomic situation of residents is more precarious. The high endemicity among under-15s in Salvador is a concern and emphasises the intensity of the transmission of *M. leprae* in certain areas of the city inhabited by populations with unfavorable life conditions.

This study has some limitations, which requires caution in the interpretation of their results. One of them refers to the quality of secondary data. In general, there is no duplication of notification because they were excluded from the information system. However, there may be underreporting of cases of leprosy leading to sub estimation of detection rates, which would mean that the epidemiological situation could be even worse than described. In addition, the profile of the disease described in this study could not be corresponding to the current epidemiological situation because the data refer to the period 2007-2011. However, it can be said that there were no major changes in this situation because the detection rate recorded in SINAN in the following period ranged from 4.8 (2014) to 7.3/100,000 inhabitants (2012) and in 2015 reached 6,0/100,000 children under 15 years (SMS 2016). Despite the small number (145) of cases of leprosy to be distributed in 157 neighborhoods, it found that cases were registered only in 44 (28%) neighborhoods located mostly in only three SD. Thus, it was possible to identify the areas of the city with the highest risk of the disease.

Regardless this limitations, the results of this study reveal the necessity to develop strategies special of prevention, painstaking and meticulous in the cities with high rate detection of leprosy, focused on the intra urban areas with hyperendemicity. In Brazil, it can be possible to do it by way the capacitation of the professionals that work in Family Health Program aiming to do active search of cases, through examination of skin and neurological, during household visits in all people, independent if there is or not a case of leprosy in house. The objective of these interventions is increasing the detection rate of suspected cases and, consequently, increasing rates of early diagnosis and treatment, thus minimising the complications resulting from progression of this disease, sterilise the infection sources and, consequently, to produce impact in the prevalence of this disease. These initiatives obligatorily include improvements in the coverage and quality of the activities conducted within the primary healthcare network.
